# Exploring novel therapeutic strategies: Could psychedelic perspectives offer promising solutions for Alzheimer’s disease comorbidities?

**DOI:** 10.1080/19585969.2025.2480566

**Published:** 2025-03-19

**Authors:** Michael Davidson, Gabriela-Dumitrita Stanciu, Jonathan Rabinowitz, Ilinca Untu, Romeo-Petru Dobrin, Bogdan-Ionel Tamba

**Affiliations:** a University of Miami School of Medicine, Miami, FL, USA; b Advanced Research and Development Center for Experimental Medicine ‘Prof. Ostin C. Mungiu’ CEMEX, ‘Grigore T. Popa’ University of Medicine and Pharmacy of Iasi, Iasi, Romania; c Bar Ilan University, Ramat Gan, Israel; d Department of Medicine III, Grigore T. Popa University of Medicine and Pharmacy of Iasi, Iasi, Romania; e Institute of Psychiatry ‘Socola’, Iasi, Romania; f Department of Pharmacology, Clinical Pharmacology and Algesiology, ‘Grigore T. Popa’ University of Medicine and Pharmacy of Iasi, Iasi, Romania

**Keywords:** Alzheimer’s disease, comorbidities, neuroplasticity, psychedelic therapy, comprehensive care

## Abstract

The increasing prevalence of dementia within an ageing global population, combined with prolonged life expectancy, accentuates Alzheimer’s disease (AD) as a multifaceted healthcare challenge. This challenge is further compounded by the limited therapeutic options currently available. Addressing the intricacies of AD management, the mitigation of comorbidities has emerged as a pivotal facet of treatment. Comorbid conditions, such as neurobehavioral symptoms, play a role in shaping the clinical course, management, and outcomes of this pathology; highlighting the importance of comprehensive care approaches for affected individuals. Exploration of psychedelic compounds in psychiatric and palliative care settings has recently uncovered promising therapeutic potential, enhancing neuroplasticity, emotional processing and connection. These effects are particularly relevant in the context of AD, where psychedelic therapy offers hope not only for mitigating core symptoms but also for addressing the array of comorbidities associated with this condition. The integration of this comprehensive method offers a chance to significantly enhance the care provided to those navigating the intricate landscape of AD. Therefore, the current paper reviews the intricate link between more frequent additional health conditions that may coexist with dementia, particularly in the context of AD, and explores the therapeutic potential of psychedelic compounds in addressing these concurrent conditions.

## Introduction

Alzheimer’s disease (AD), the leading neurodegenerative disorder worldwide, is characterised by a progressive decline in cognitive function and neurodegeneration. It remains a significant challenge for global healthcare, particularly as the incidence of dementia rises with an ageing population and increasing life expectancies (GBD 2019 Dementia Forecasting Collaborators 2022). Projections indicate that by 2050, the global population aged 60 and over will double, expanding from 1 billion to 2 billion, with those aged 80 and above expected to triple during the same period (World Health Organization 2024). This demographic shift presents an unprecedented challenge for healthcare systems, as ageing is the most significant risk factor for AD. Furthermore, ageing is often accompanied by an increased prevalence of chronic, debilitating conditions, with depression emerging as a notable comorbidity due to its particularly poor prognosis.

Despite more than a century since AD was first identified and the amyloid hypothesis was proposed, a cure or well-established disease-modifying treatment remains elusive, even after extensive research and over 200 failed candidate drugs (Dubois et al. 2014). The amyloid hypothesis has long been the dominant framework in AD research, largely due to the strong correlation observed between beta-amyloid (Aβ) plaques and the hallmark pathological features of the disease, including neuronal loss and cognitive decline. According to this hypothesis, the accumulation of Aβ plaques in the brain is thought to play a central role in triggering the neurodegenerative processes that lead to the clinical manifestation of AD. However, the dominance of the amyloid hypothesis has been increasingly questioned due to mounting evidence that Aβ accumulation alone may not fully explain the complexity of AD pathogenesis (Hardy and Selkoe, 2002; Sharma 2019). Furthermore, clinical trials targeting amyloid plaques have largely failed to demonstrate significant long-term cognitive improvements, highlighting the limitations of amyloid-based approaches.

Current therapeutic strategies primarily target memory enhancement through acetylcholinesterase inhibitors, which provide modest benefits for certain patients (Sharma 2019; Stanciu et al. 2019; Marucci et al. 2021). However, these treatments do not modify or slow the underlying neurodegenerative processes, and their benefits tend to be short-lived, particularly in the later stages of the disease. Recently, several novel antibody-based therapies, such as Aducanumab and Donanemab, have been developed to directly target and clear amyloid plaques (Sevigny et al. 2016; Mintun et al. 2021; Ayton 2021). While these therapies have shown some success in reducing amyloid burden, their clinical efficacy has been inconsistent, with only modest improvements observed in cognitive function and limited benefits across diverse patient populations (Sevigny et al. 2016; Mintun et al. 2021; Ayton 2021). Moreover, these treatments have raised significant concerns regarding their safety profiles, with side effects such as brain swelling (amyloid-related imaging abnormalities) and micro-hemorrhages, as well as their high cost, which presents substantial barriers to widespread clinical implementation (Tampi et al. 2022; Lacorte et al. 2022). These factors have sparked considerable debate within the scientific community regarding the clinical utility of these therapies and their potential role in the broader landscape of AD treatment.

Neurodegenerative disorders like AD are often accompanied by a range of comorbidities, which may either serve as risk factors for the development of the disease or result from its ongoing pathological processes. AD predominantly affects individuals aged 65 and older, many of whom also suffer from comorbid conditions such as diabetes, hypercholesterolaemia, atherosclerosis, hypertension, obesity, stroke, and chronic kidney disease (Stanciu et al. 2020; Stanciu et al. 2021). These conditions can act as triggers or accelerators of the neuropathological features associated with AD, particularly the formation and accumulation of Aβ plaques and neurofibrillary tangles (NFTs). Alongside cognitive decline, patients with AD often experience various neuropsychiatric symptoms, including agitation, depression, apathy, sleep disturbances, and anxiety (Steinberg et al. 2008; Botto et al. 2022). These symptoms can lead to increased disability, a decline in quality of life, challenges in daily activities, heightened caregiver burden, a greater risk of institutionalisation, and elevated mortality rates (Soto et al. 2015). Although the link between AD and its comorbidities remains partially understood, emerging evidence highlights the significant role of systemic dysfunctions—particularly chronic inflammation, insulin resistance, and vascular impairments—in accelerating disease progression. The role of chronic inflammation in AD is particularly evident in comorbidities like obesity, diabetes, and cardiovascular disease, which all contribute to a state of low-grade, systemic inflammation. This chronic inflammatory response, often referred to as inflammaging, exacerbates the deposition of Aβ and tau tangles, further impairing cognitive function (Zhang et al. 2023). The systemic release of pro-inflammatory cytokines, such as TNF-α and IL-1β, can directly affect brain tissue, impairing neuronal function and promoting neurodegenerative changes (Zhang et al. 2023). In addition to inflammation, insulin resistance plays a significant role in AD progression. Insulin resistance, commonly associated with type 2 diabetes, has been linked to impaired glucose metabolism in the brain, reduced synaptic plasticity, and an increased risk of Aβ accumulation (Ferreira et al. 2018). This metabolic dysfunction, when coupled with the neurodegenerative processes of AD, leads to a vicious cycle that accelerates cognitive decline. Furthermore, vascular comorbidities, including hypertension and stroke, contribute to reduced cerebral blood flow and damage to the blood-brain barrier, further exacerbating Aβ deposition and neuronal loss (Iadecola 2013). Given the complex interplay between these comorbid conditions and the underlying neurodegeneration in AD, there is an increasing need to address not only the core symptoms of the disease but also the comorbidities that complicate its progression. Emerging research suggests that psychedelic compounds, which act on serotoninergic systems and promote neuroplasticity, may offer a novel approach for addressing both the cognitive and ­neuropsychiatric symptoms of AD, as well as its comorbidities.

Psychedelic substances, through their interaction with serotonin and dopamine receptors, particularly the 5-HT2A receptor, have been shown to enhance structural and functional neuroplasticity (Inserra et al. 2021). Furthermore, they possess significant anti-inflammatory properties, which could help modulate the systemic inflammation associated with both AD and its comorbidities (Flanagan and Nichols 2018; Preller et al. 2020; Hutten et al. 2021). By targeting neuroplasticity and inflammation, psychedelics may not only alleviate cognitive decline but also help mitigate the impact of comorbid conditions such as diabetes and cardiovascular disease, offering a more integrated approach to AD treatment. Additionally, these substances exhibit anti-addictive, antidepressant, and anxiolytic properties (Garcia-Romeu et al. 2016). This integrated therapeutic strategy could significantly improve patient outcomes by addressing the multifaceted nature of the disease, both in terms of neurodegeneration and comorbidity management. Accordingly, this paper reviews the intricate relationships between most prevalent health conditions that may coexist with dementia, especially in relation to AD, and explore the therapeutic potential of psychedelic compounds for these concurrent conditions.

## Physiological and behavioural effects of psychedelics relevant to Alzheimer’s disease

Cognitive decline is one of the key neuropsychiatric symptoms of AD, significantly impacting patients’ quality of life. A key player in many behavioural and cognitive processes is serotonin (5-hydroxytryptamine, 5-HT), a neurotransmitter involved in mood regulation, perception, anxiety, aggression, and appetite. Notably, the 5-HT2A receptor, which is highly concentrated in brain regions such as the prefrontal cortex and hippocampus—areas particularly affected in AD—plays a critical role in these functions. Psychedelics, which include compounds like psilocybin, D-lysergic acid diethylamide (LSD), dimethyltryptamine (DMT), mescaline and cannabis (especially tetrahydrocannabinol – THC), interact with these serotonin receptors, especially 5-HT2A (Carhart-Harris and Goodwin 2017). Although often labelled as ‘hallucinogens’, the effects of these substances extend far beyond hallucinations, influencing a wide range of cognitive and perceptual processes. Psychedelics may offer an alternative treatment for AD, focusing on their ability to enhance neuroplasticity and modulate key neurotransmitter systems (Table 1).

**Table 1.** Potential neuropsychological effects of psychedelics relevant to Alzheimer’s disease.

Neuromechanisms	Molecular target and key signalling pathways	Findings	References
Structural and functional neuroplasticity	Serotonin 5-HT2A receptors	A single dose of psilocybin (1 mg/kg) in mice resulted in rapid and persistent growth of dendritic spines in the frontal cortex and ameliorated stress-related behavioural deficits.	Shao et al. (2021)
		Single low doses of LSD (5, 10, and 20 μg) in healthy volunteers lead to the activation of AMPA receptors and increased expression of BDNF	Hutten et al. (2021), Mason et al. (2020)
		A dose of 3.5 μg/ 35 g of psilocybin increased the formation of neurons in the dentate gyrus of mice. Repeated intraperitoneally administration of 52 μg/35 g psilocybin enhanced neuroplasticity	Catlow et al. (2013, 2016)
		Chronic administration in rats of ayahuasca 150 mL/70 kg resulted in increased in BDNF	Colaço et al. (2020)
		A single dose of 2 mg/kg DOI increased dendritic structural density and plasticity in the prefrontal cortex and enhance long-term potentiation in mice	Revenga et al. (2021)
		Chronic treatment C57Bl/6 mice with DOI, TCB-2 and 25CN-NBOH (1 mg/kg, 14 days) produced increased pro-BDNF levels and downregulation of TrkB receptors.	Tsybko et al. (2020)
		A single dose of psilocybin (0.08 mg/kg) increased the density of hippocampal synaptic vesicle protein 2 A	Raval et al. (2021)
		A single dose of DOI, DMT, and LSD (1, 10 mg/kg) selectively increased neuritogenesis, spinogenesis, and synaptogenesis both *in vitro* and *in vivo via* the modulation of the mTOR and TrkB	Ly et al. (2018)
Cognitive effects	Serotonin 5-HT2A receptors	Micro-doses of psilocybin (0.22, 0.33, or 0.44 g of dried truffle) were associated with improvements in cognitive fluency, flexibility, and originality in healthy adults	Prochazkova et al. (2018)
		In a rat model of depression, repeated doses of LSD (0.13 mg/kg) have been found to enhance both prospective and retrospective learning	Buchborn et al. (2014)
		A single dose of LSD (0.13 mg/kg) enhanced novel object preference in young and adult rats	Cini et al. (2019)
		Psilocybin therapy, administered in two sessions (20 mg/70 kg in session 1 and 30 mg/70 kg in session 2), enhances cognitive and neural flexibility in both healthy adults and individuals with major depressive disorder.	Doss et al. (2021); Nayak et al. (2023)
		In healthy volunteers, a dose of 0.17 mg/kg psilocybin decreases convergent thinking and enhances both spontaneous and goal-directed divergent thinking	Mason et al. (2019, 2021)
	mGluR2 receptor	Inhibition of the mGluR2 receptor in the frontal neurons of rats resulted in reduced cognitive flexibility and increased alcohol-seeking behaviour. Treatment with psilocybin at doses of 1 mg/kg and 2.5 mg/kg mitigated these deficits	Meinhardt et al. (2021)
Neuroinflammation	Serotonin 5-HT2A receptors Sig-1R receptors	DOI (0.1 pM to 100 nM in cell culture; 0.1 µg/kg and 0.3 µg/kg in mice), LSD (3.5 nM or 15 nM concentrations), DMT (100 µM), and 5-MeO-DMT (100 µM) determined the inhibition of IL-1β, IL-6, and TNF-α in cellular or *in vivo* models	Yu et al. (2008); Nau et al. (2013); Szabo et al. (2014)
		A single 1 mL/kg oral dose of DMT in an ayahuasca admixture reduced C-reactive protein levels and correlated with mood improvements in treatment-resistant depression	Galvão-Coelho et al. (2020)
		Psilocybin and 4-AcO-DMT (10, 20, and 40 μM) attenuates TNF-α and IFN-γ-induced inflammation in human small intestinal epithelial cells by reducing COX-2 and IL-6 expression, without affecting cell viability. Consistent anti-inflammatory effects were observed in animal models and a 3D human intestinal model, marked by decreased levels of TNF-α, IFN-γ, IL-6, and IL-8	Robinson et al. (2023)
		At a dosage of 0.17 mg/kg, psilocybin decreases the concentrations of TNF-α, IL-6, and C-reactive protein in healthy individuals	Mason et al. (2023)
		Psilocybin treatment (0.88 mg/kg) reduces the expression of several markers, including COX-2, TNF-α, IL-1β, IL-6, and IL-8, in a mouse model of brain inflammation induced by LPS	Zanikov et al. (2023)

5-HT_2A_R receptor, serotonin 2 A (5-HT_2A_) receptor; AMPA receptors, α-amino-3-hydroxy-5-methyl-4-isoxazolepropionic acid receptor; BDNF expression, brain-derived neurotrophic factor expression; LSD, lysergic acid diethylamide; psychedelic DOI, psychedelic 1-(2,5-dimethoxy-4-iodophenyl)-2-aminopropane; TCB-2, 4-Bromo-3,6-dimethoxybenzocyclobuten-1-yl) methylamine hydrobromide; TrkB receptors, tropomyosin receptor kinase B; 25CN-NBOH, 4-[2-[[(2-Hydroxyphenyl)methyl]amino]ethyl]-2,5-dimethoxybenzonitrile hydrochloride; mTOR, mammalian target of rapamycin; Sig-1R, opioid-like orphan receptor sigma-1 receptor; nM, nanomolar; pM, picomolar; 5-MeO-DMT, 5-methoxy-N,N-dimethyltryptamine; 4-AcO-DMT, 4-Acetoxy-N,N-dimethyltryptamine, also known as O-Acetylpsilocin; COX-2, cyclooxygenase-2; TNF-α, tumour necrosis factor-alpha; L-1β, interleukin-1 beta; IL-6, interleukin-6; IL-8, interleukin-8

Neuroplasticity plays a crucial role in cognitive function, particularly in neurodegenerative diseases like Alzheimer’s, where synaptic loss and neuronal atrophy contribute to cognitive decline. Psychedelics, through the activation of 5-HT2A receptors, have been shown to enhance synaptogenesis, dendritic remodelling and neurogenesis, primarily *via* mTOR and BDNF signalling pathways. These mechanisms are essential for maintaining and restoring neural connectivity, particularly in brain regions affected by AD, such as the prefrontal cortex and hippocampus. Preclinical and clinical studies suggest that psilocybin and related compounds can improve cognitive flexibility, learning, and memory by reinforcing synaptic integrity and facilitating adaptive neural responses. By counteracting synaptic deterioration, psychedelics may offer a therapeutic strategy to slow cognitive decline and enhance cognitive function in Alzheimer’s patients, bridging the gap between molecular mechanisms and clinical outcomes (Doss et al. 2021; Shao et al. 2021). Beyond their cognitive benefits, psychedelics also exert significant effects on mood and behaviour through the serotonergic system. By activating 5-HT2A receptors and modulating glutamatergic pathways, psilocybin has demonstrated anxiolytic and antidepressant properties, especially in patients with major depression (Catlow et al. 2013; Carhart-Harris and Goodwin 2017). These same serotonergic mechanisms may help reduce neuropsychiatric symptoms commonly seen in Alzheimer’s disease, such as anxiety, depression, and agitation. As a result, these compounds could significantly enhance the psychological well-being and overall quality of life for dementia patients (Mason et al. 2019, 2020, 2021). Chronic neuroinflammation is a critical contributor to Alzheimer’s disease pathology, exacerbating neuronal damage and accelerating beta-amyloid accumulation. The ability of psychedelics to reduce neurogenic inflammation ­provides another significant benefit. This is achieved by inhibiting pro-inflammatory cytokines such as TNF-α and IL-6, while also modulating Sig-1R receptors, which contribute to their neuroprotective effects (Yu et al. 2008; Nau et al. 2013; Szabo et al. 2014). Clinical and preclinical evidence suggest that psychedelics can mitigate neuroinflammation, thus preserving neuronal health. For Alzheimer’s patients, this anti-inflammatory action is crucial, as it can protect neurons from degeneration and potentially slow the progression of cognitive decline (Robinson et al. 2023; Mason et al. 2023). Reducing neuroinflammation could, therefore, be an important therapeutic strategy in neurodegenerative diseases. Beyond their direct impact on cognition and neuroinflammation, psychedelics may also address a range of comorbid symptoms frequently observed in Alzheimer’s disease. These include neuropsychiatric disturbances as well as metabolic and cardiovascular conditions such as obesity, diabetes, cardiovascular disorders, and pain, all of which contribute significantly to patient distress and caregiver burden (van Dongen et al. 2021; Rosenblat et al. 2024; Gojani et al. 2024). By leveraging their ability to modulate serotonergic pathways and promote neural homeostasis, psychedelics may offer a multifaceted therapeutic approach that extends beyond cognitive benefits.

## Therapeutic role of psychedelics on Alzheimer’s disease comorbidities

### AD comorbid with neuropsychiatric symptoms

AD patients frequently experience comorbid neuropsychiatric symptoms (NPS), which are partially attributed to disruptions in the serotonergic system and recognised as contributing to disease progression (Zhao et al. 2016; Ruthirakuhan et al. 2019). Additionally, more severe symptoms are associated with an increased risk of cognitive decline, often serving as early indicators of prodromal dementia and associated with early signs of AD biomarkers (Banning et al. 2021).

A growing number of studies indicate that psychedelics may be highly effective in addressing mood, anxiety, and depression, particularly in cases resistant to conventional treatments (Carhart-Harris et al. 2016). The administration of psilocybin at repeated doses has been associated with significant reductions in depression severity, particularly among individuals diagnosed with treatment-resistant major depressive disorder (MDD). In the phase 2, randomised feasibility study conducted by Rosenblat et al. (2024), participants were adults under the age of 65 diagnosed with treatment-resistant depression, with specific exclusion criteria for individuals with psychosis or active substance use disorders. The study included one to three psilocybin sessions, each involving a fixed dose of 25 mg, with outcomes assessed over a six-month period. In a randomised clinical study involving 27 individuals aged 21 to 75 years diagnosed with major depression, participants were not currently using antidepressant medications and had no history of psychotic disorder, serious suicide attempt, or hospitalisation. The study involved two psilocybin sessions, with a fixed dose of 20 mg/70 kg for session 1 and 30 mg/70 kg for session 2. Participants who received psilocybin exhibited significantly greater reductions in depression severity compared to those in a wait-list control group, with more than half meeting the criteria for remission four weeks following treatment. The intervention period lasted 8 weeks, with outcomes assessed at multiple time points (Davis et al. 2021). Additionally, in a phase 2, open-label trial, a single 25 mg dose of psilocybin resulted in a significant reduction in depression severity scores among cancer patients with major depressive disorder (MDD) (Agrawal et al. 2024). This trial enrolled participants aged 18 years or older, including both those with curable and non-curable cancer. Eligible participants (*n* = 30) had a diagnosis of MDD (either single episode or recurrent, without psychotic features). Outcomes measured in the study included depression severity, anxiety, pain, demoralisation, and disability. At week 1, 50% of participants showed full remission of depressive symptoms, and 80% demonstrated a sustained response to treatment, with the improvements lasting for 8 weeks. In another study, individuals with treatment-resistant bipolar type II major depressive episodes demonstrated both response and remission after receiving a single 25 mg psilocybin dose (Aaronson et al. 2024). This 12-week open-label trial included 15 participants aged 18 to 65 years. Notably, a double-blind, randomised trial with 30 patients aged 18 to 80 years showed that individuals with MDD who received three doses of 25 mg psilocybin exhibited personality changes indicative of improved mental health. The study was conducted over a core 6-week trial period (Weiss et al. 2024).

Moreover, a randomised, double-blind, cross-over trial investigated the effects of psilocybin on 51 cancer patients aged 21 to 80 years, all of whom had life-threatening diagnoses and symptoms of depression and/or anxiety, with no central nervous system (CNS) involvement disorders. The study compared the effects of a very low (placebo-like) dose (1 or 3 mg/70 kg) versus a high dose (22 or 30 mg/70 kg) of psilocybin, administered in a counterbalanced sequence with a 5-week interval between sessions. As a result, a single high dose of psilocybin can elicit clinically significant reductions in depressive and anxiety symptoms among patients with life-threatening cancer diagnoses (Griffiths et al. 2016). Another rigorously designed study evaluated the comparative efficacy of two doses of psilocybin (1 mg and 25 mg) against daily escitalopram (10 mg for the first week, then increasing to 20 mg) over a six-week period in a cohort of 59 participants with moderate to severe major depression. Although both treatment groups demonstrated significant symptom alleviation, psilocybin was associated with superior overall improvement, evidenced by a higher remission rate (57% vs. 28%) (Carhart-Harris et al. 2021).

In addition to these clinical findings, preclinical studies further support the potential antidepressant and anxiolytic effects of psychedelics. Both psilocybin and DMT have been shown to facilitate fear extinction in murine models, whether administered as a single high dose or in repeated low doses, while also inducing long-lasting antidepressant-like effects, as evidenced by forced swim tests (Catlow et al. 2013; Cameron and Olson 2018; Cameron et al. 2019; Hibicke et al. 2020). Furthermore, psilocybin has been found to mitigate stress-induced anhedonia, indicated by increased preference for sucrose and heightened responsiveness in female urine sniffing tests (Hesselgrave et al. 2021). It also significantly reduces escape failures in learned helplessness models, ­suggesting a decrease in depressive behaviours. Collectively, these findings underscore the potential of psychedelics as a viable therapeutic alternative, demonstrating efficacy that may equal or surpass that of conventional selective serotonin reuptake inhibitors (SSRIs) in the management of depressive disorders (Shao et al. 2021).

In the context of current clinical investigations, there is only one ongoing pilot study evaluating the potential of psilocybin for NPS in patients with early-stage AD and mild cognitive impairment (MCI). This trial is the first to investigate both moderate (15 mg/70 kg) and high doses (25 mg/70 kg) of psilocybin in patients with these conditions who also experience depressive symptoms (ClinicalTrials.gov NCT04123314). In contrast, several randomised controlled trials have explored the efficacy of THC and its derivatives in similar contexts. Existing research indicates that low-dose of THC may be effective in treating agitation, aggression, irritability, emotional lability, anxiety, and insomnia (Sawicki et al. 2023). On the other hand, a third trial, which focused on nabilone, suggested its potential efficacy in alleviating agitation, provided that careful monitoring of sedation and agitation is maintained to promote reductions in anxiety and depressive symptoms in patients with moderate-to-severe AD (Ruthirakuhan et al. 2019). The observed decrease in anxiety and depression is particularly significant for AD individuals, as NPS are often correlated with a decline in quality of life. Therefore, further investigation is necessary to clarify how psychedelics influence emotional and psychological outcomes in AD patients, which is vital for creating more targeted and effective therapeutic strategies for neuropsychiatric symptom management.

### AD comorbid with obesity and diabetes

In AD, impaired glucose uptake in the brain is a key factor contributing to cognitive decline. Similarly, obesity is often characterised by insulin resistance and disrupted glucose metabolism. Psychoactive drugs have demonstrated potential in enhancing brain function by improving glucose utilisation and uptake (Dwyer et al. 2002). This suggests that these drugs could provide dual benefits in managing both AD and obesity-related metabolic issues. Recent studies have explored the effects of psilocybin (10 μM) on pancreatic β-cell health under conditions of high glucose and lipids. Research indicates that psilocybin can mitigate β-cell loss caused by these metabolic stressors. This protective effect is thought to be mediated by modulating apoptotic pathways, including the phosphorylation of TXNIP, STAT-1, and STAT-3. Additionally, psilocybin influences the expression of genes related to β-cell dedifferentiation, such as Pou5f1 and Nanog, suggesting potential for attenuating β-cell dedifferentiation and supporting further investigation into its therapeutic applications for type 2 diabetes (Gojani et al. 2024). Complementing these findings, the only existing study using 18-fluorodeoxyglucose-PET, in healthy volunteers, has shown that a single dose of psilocybin (15 mg or 20 mg) results in an approximately 25% increase in global brain glucose metabolism, particularly in the frontal and medial temporal cortices (Vollenweider et al. 1997). Furthermore, psychedelics have been shown to enhance cognitive function and mood, which may offer indirect benefits for AD patients also grappling with obesity-related issues. As research into psychedelics advances, these substances could provide novel approaches for addressing the complex interplay between AD, obesity, and diabetes, underscoring the need for further investigation into their therapeutic potential.

### AD comorbid with cardiovascular disease

Psychedelics such as psilocybin, mescaline, and LSD act as agonists of serotonergic receptors, leading to vasoconstriction and increased blood pressure, with LSD showing the strongest effect, especially at higher doses (Schlag et al. 2022). This vasoconstriction can result in severe vasospasm, as observed in various clinical case studies of non-medical intoxication (Nichols 2016). Serotonergic hyperactivity is also linked to an increased risk of developing pulmonary arterial hypertension (PAH), though direct research on the effects of psychedelics on pulmonary pressure remains limited (Aghajanian and Marek 1999). Given serotonin’s role in the pathophysiology of PAH, further investigation into the safety of psychedelic use in patients with pulmonary hypertension is warranted.

Psychedelics may also increase the risk of thromboembolic complications, as serotonin can promote platelet aggregation *via* 5-HT2A receptors (McClue et al. 1989). Although this raises concerns about the potential for blood clots, serious thromboembolic events have mostly been reported in situations of drug abuse rather than in controlled therapeutic settings (Borowiak et al. 1998). The effects on cardiac function are complex and variable. For example, psilocybin-containing mushroom extracts have shown some protective effects against heart cell injury in experimental models, such as reducing cell damage and inflammation (Nkadimeng et al. 2020). However, high doses of psilocin can cause issues like tachycardia, myocardial ischaemia, and conduction abnormalities (Borowiak et al. 2006). LSD, when tested in cardiac tissues, has been found to increase contractility and heart rate through its interaction with serotonin 5-HT4 and histamine H2 receptors, with these effects being dose-dependent (Gergs et al. 2024).

Additionally, psychedelics may influence the BDNF-TrkB signalling pathway, which is important for neuroplasticity and could have implications for cardiovascular health, as BDNF is synthesised in cardiac tissues and influences cardiac function and inflammation (Donovan et al. 2000). This suggests a potential role for psychedelics in modulating cardiac health, but further research is needed to understand the implications fully. Developing animal models to study these effects has proven challenging due to species-specific differences in cardiovascular responses, making it difficult to predict how these findings translate to human outcomes. As a result, while psychedelics show potential for therapeutic use, particularly in neuropsychiatric contexts, their cardiovascular effects need thorough investigation to ensure safe application in individuals with underlying cardiovascular risks. The combined effects of psychedelics on AD patients with coexisting cardiovascular disease are not well-studied. Most research so far focuses either on the cognitive benefits for neurodegenerative diseases or the risks for those with cardiovascular conditions separately. Long-term studies are needed to assess the safety, efficacy, and potential interactions between psychedelics and the cardiovascular drugs often prescribed to these patients.

### AD comorbid with chronic pain

The comorbidity of AD and chronic pain is a complex issue that has gained attention due to the overlapping mechanisms and challenges in managing both conditions simultaneously. Chronic pain is particularly challenging to manage in AD patients because cognitive deficits can complicate communication about pain levels, making it harder for caregivers and clinicians to accurately assess and address their pain.

Recent research has investigated the use of psychedelics like psilocybin and LSD for various pain conditions, including cluster headaches, migraines, fibromyalgia, chronic back pain, and neuropathic pain. Studies by Bornemann et al. (2021) and Bonnelle et al. (2022) have highlighted that both micro- and macro-doses of these psychedelics can effectively alleviate pain for up to six months, with superior results compared to nonsteroidal anti-inflammatory drugs. Although macro-doses seemed to offer longer-lasting relief than microdoses, this difference was not statistically significant.

Research on psychedelic use for cancer pain is ­limited, with only two key reports available. The first, a study by Kast and Collins (1964), found that 100 μg of LSD-25 produced more sustained pain relief compared to traditional opioids like hydromorphone and meperidine. Participants treated with LSD-25 experienced notably longer periods without pain—averaging 95.6 min pain-free compared to 8.4 and 5.7 min for hydromorphone and meperidine, respectively. Additionally, almost half (48.9%) of those receiving LSD-25 remained pain-free for over 19 h post-treatment, whereas no patients in the comparison groups achieved such prolonged relief. A subsequent case study described symptom improvement in a patient with depression and multiple myeloma after MDMA administration (50–150 mg) (Greer and Tolbert 1998). Both LSD-25 and MDMA thus show potential for managing pain in cancer patients, although more research is needed to confirm these findings.

Additionally, studies on the physiological effects of psychedelics in healthy participants and recreational users have shown mixed outcomes. Psilocybin, in doses up to 30 mg/70 kg, induced mild to moderate headaches in a dose-dependent manner (Johnson et al. 2003), while a 20 µg dose of LSD provided pain relief without substantial cognitive or physical disruptions during cold-pressor tests. Chronic use of MDMA (3,4-methylenedioxymethamphetamine) has been associated with increased negative outcomes, such as reduced pain tolerance and a decline in mood among recreational users compared to non-users (O’Regan and Clow 2004). Additionally, symptoms like ‘visual snow’, characterised by persistent visual disturbances such as palinopsia and sensitivity to light, have been more frequently observed (van Dongen et al. 2021). It is important to note that these studies also included participants using various other illicit substances, which may have influenced the findings and added complexity to the interpretation of MDMA’s specific effects.

There are no direct clinical studies that specifically investigate the use of psychedelics in patients who have both AD and chronic pain. Most of the existing research is either focused on the potential cognitive benefits of psychedelics in AD or their pain-relieving effects in other conditions. However, there are some preclinical studies and early-phase trials that suggest psychedelics could have a dual benefit due to their neuroplastic and analgesic properties. For example, the potential to enhance neuroplasticity in AD could theoretically benefit cognitive function, while the analgesic effects might alleviate chronic pain symptoms.

## Conclusions and prospects

Currently, there is limited data regarding the regulation and therapeutic use of psychedelics, such as psilocybin, for Alzheimer’s disease (AD) and its associated comorbidities. However, psychedelics have demonstrated neuroplasticity-promoting effects, which may counteract synaptic loss and neuronal dysfunction characteristic of AD. Additionally, their ability to modulate neuroinflammation through immunomodulatory properties offers a promising mechanism for addressing one of the key drivers of AD progression. The potential therapeutic applications extend beyond AD itself, as psychedelics may also provide benefits in managing common comorbidities such as depression and anxiety, which frequently accompany neurodegenerative diseases.

Randomised clinical trials investigating the efficacy of psychedelics in neuropsychiatric symptoms have shown promising antidepressant and anxiolytic effects. These findings suggest that psychedelics could be leveraged to address mood disturbances, anxiety, and agitation in AD patients. The multifaceted pharmacological profile of psychedelics, including their impact on serotonin receptors and glutamatergic pathways, positions them as a unique alternative to conventional treatments, offering potential advantages over single-target therapies.

However, while these compounds present exciting opportunities, there remain several important safety concerns, particularly for elderly patients with AD and other neurodegenerative diseases. Key safety issues include the potential exacerbation of pre-existing neuropsychiatric symptoms such as anxiety and psychosis (Carhart-Harris and Goodwin 2017), which may be triggered by the serotonergic effects of psychedelics (Krebs and Johansen 2013; Nichols et al. 2017). Furthermore, the cardiovascular effects of psychedelics, such as increased blood pressure and heart rate, warrant caution in elderly individuals with common comorbidities such as hypertension or heart disease (Wsol 2023). It is essential that clinical studies specifically address these concerns, carefully monitoring the risks associated with psychedelics use in vulnerable populations.

Despite these challenges, early-phase studies show encouraging results regarding the safety and efficacy of psychedelics in alleviating neuropsychiatric symptoms and improving quality of life in patients with depression associated with neurodegenerative conditions. However, many of these studies are limited by small sample sizes and short follow-up periods, which restrict the ability to draw definitive conclusions (Krebs and Johansen 2013; Griffiths et al. 2016; Zheng et al. 2024). Emerging research also hints at the potential neuroprotective effects of psychedelics (Garcia-Romeu et al. 2022), but further investigation is required to determine their long-term impact on AD progression.

Looking ahead, more extensive randomised controlled trials are essential to evaluate not only the efficacy of psychedelics in treating neuropsychiatric and cognitive symptoms but also their impact on disease progression in AD patients. Special attention should be given to identifying patient subtypes that may benefit the most, as well as the long-term effects of treatment. Ethical and regulatory considerations will play a crucial role in advancing these therapies, requiring rigorous safety protocols and clear guidelines for their clinical use. While the current body of literature is promising, further research is needed to validate the therapeutic potential of psychedelics for AD and to establish a solid foundation for their integration into clinical practice.
